# Blindness after laryngectomy and bilateral neck dissection in a diabetic patient: case report

**DOI:** 10.1590/S1516-31802001000500006

**Published:** 2001-09-01

**Authors:** Rui Celso Martins Mamede, David Livingstone Alves Figueiredo, Fabrício Villela Mamede

**Keywords:** Blindness, Neck dissection, Complications of neck dissection, Complications of Diabetes Mellitus, Amaurose, Esvaziamento Cervical, Complicações dos Esvaziamentos Cervicais, Complicações dos *Diabetes Mellitus*

## Abstract

**CONTEXT:**

Neck dissection that accompanies resection of the primary lesion in malignant tumors of the upper aerodigestive tracts may cause complications inherent to the procedure or to prolongation of surgical time, increasing the risks for the patient. Among the complications that might occur is blindness, a rare complication with only 10 cases reported in the literature thus far.

**OBJECTIVE:**

To present the case of a diabetic patient submitted to total laryngectomy and modified and selective neck dissection that resulted in blindness.

**CASE REPORT:**

The authors report on a patient submitted to total laryngectomy and selective neck dissection on the left side, and modified radical neck dissection on the right, who developed blindness. This was probably due to intraoperative hypotension plus the contribution of decompensated diabetes mellitus and thrombosis of the internal jugular vein on the right side. The possible causes, risk factors and care to be taken to prevent this rare but highly debilitating complication are discussed.

## RESUMO

**CONTEXTO:**

O esvaziamento cervical que acompanha a ressecção da lesão primária no tratamento dos tumores malignos das vias aerodigestivas superiores possibilita o surgimento de complicações inerentes a ela ou ao prolongamento do tempo cirúrgico, aumentando os riscos para o paciente. Entre as complicações que podem ocorrer está a amaurose, de incidência rara.

**RELATO DO CASO:**

Um paciente submetido a laringectomia total e esvaziamento cervical seletivo à esquerda e radical modificado à direita, que evoluiu com amaurose, provavelmente decorrente de hipotensão intra-operatória, com contribuição dos *Diabetes Mellitus* descompensado e trombose da veia jugular interna à direita. Discutem as possíveis causas, os fatores de risco e os cuidados que devem ser tomados para evitar essa rara, mas tão debilitante complicação.

## Introduction

Neck dissection that accompanies resection of the primary lesion in the treatment of malignant tumors of the upper aerodigestive tracts is a very important intervention in terms of prognosis. The importance of this technique comes from the very high frequency of invasions of the lymphatic system by squamous cell carcinomas, which are the most common types of head and neck tumors.^1,2^ When neck dissection is added to the removal of the primary lesion, complications inherent to it or to the prolongation of the surgical act are introduced, increasing the risks for the patients. Among the complications that might occur is blindness, a rare complication with only 10 cases reported in the literature thus far.^3^

The objective of this report is to present the case of a diabetic patient submitted to total laryngectomy and modified and selective neck dissection that resulted in blindness.

## CASE REPORT

A 54-year-old white male patient, a smoker (10 cigarettes/day/15 years), was admitted to our service on March 26, 1998 with a complaint of dysphonia of 3 months duration associated with earache on the right side. The patient denied having dysphagia/odynophagia, dyspnea or other complaints. Clinical and radiological examination including video-laryngoscopy revealed a lesion with neoplastic characteristics localized in the supraglottis on the right side, with T_3_N_0_M_x_ clinical staging. During hospitalization, blood glucose levels ranged from 200 to 280 mg/dl. Preoperative hemoglobin and hematocrit were 12 and 36% respectively, arterial pressure was 130 x 70 mm Hg, and heart rate 80 bpm.

The patient was submitted to total laryngectomy and lateral neck dissection on the left side, and modified type III radical neck dissection on the right side. During the operation, ganglia suspected of containing metastases were observed. Surgery lasted approximately 7 hours, with intraoperative transfusion of 3000 ml of crystalloids, 460 ml of plasma and 300 ml of red blood cell concentrate. Intraoperative arterial pressure was 100 x 60 mmHg, with episodes of hypotension of approximately 40 minutes during which arterial pressure was 80 x 45 mmHg. Intraoperative urinary output was 225 ml over the 7-hour period. Postoperative tests revealed hemoglobin = 10.3 and hematocrit = 32.6%. Between the 4^th^ and 5^th^ postoperative day, the patient started to complain of significant visual clouding, which progressed to blindness. Ophthalmological evaluation revealed the presence of considerably reduced intrinsic reflexes, with cloudy papillae and edema of the optic disk. Bilateral anterior ischemic optic neuritis was diagnosed. No visual improvement occurred over the subsequent days.

## DISCUSSION

The literature has occasionally reported cases of blindness as a complication of cardiovascular surgeries, general surgeries, and especially ophthalmological procedures,^2^ but few reports are available about blindness as a complication in patients submitted to neck dissection. In the few cases cited in the literature, there were doubts about whether the surgical act was responsible for blindness or another motive was involved.

In the light of physiopathology, two mechanisms can explain sudden blindness, i. e. venous congestion and ischemia. Ligation of the jugular veins may cause an increase in central venous pressure and cerebrospinal fluid pressure, with consequent venous congestion. This is the most accepted mechanism for explaining the blindness of patients submitted to radical neck dissection. However, when cerebrospinal fluid pressure is monitored, it is found to be increased during dissection and only for a period of 24-48 hours thereafter,^3^ i.e. by 24-48 hours after the procedure the pressure has already normalized. Thus, this does not explain why blindness should occur on the 5^th^ postoperative day, as observed in the case presented here. Furthermore, pressure monitoring has revealed that the increase in cerebrospinal fluid pressure occurring when the jugular vein is ligated bilaterally does not differ from the increase observed when the ligature is unilateral.^3^ Another important aspect that raises doubts about this hypothesis is that the position of the patient's head alters cerebrospinal fluid pressure more than bilateral ligation of the jugular veins does. This fact would suggest that cases of blindness ought to be more frequent and independent of the type of neck dissection.^3^

The blindness in our patient could only be explained by alteration in cerebrospinal fluid pressure due to head positioning, since he was submitted to modified neck dissection on one side and to selective neck dissection on the other. These are procedures that do not require ligation of the jugular veins.

Another situation that might provoke blindness secondary to venous congestion among patients submitted to neck dissection with preservation of the internal jugular veins is thrombosis of these veins. In our patient, Doppler ultrasound identified thrombosis of the internal jugular vein only on the right side, thus in itself not providing an explanation for the blindness on the left side. According to Larkin,^4^ increased CO_2_ concentration may also cause an increase in central venous pressure, but this variable was not measured in our patient.

Ischemia may be caused by local or systemic factors that alter the perfusion pressure of the posterior ciliary arteries, which are branches of the ophthalmic artery that supply the optic nerves. Among local factors are stenosis or spasm of the ciliary artery, ophthalmic artery or carotid artery provoked by atherosclerotic disease, hypertension, collagen disease, temporal arteritis or diabetes. Among the systemic causes are arterial hypotension and hematological disorders such as polycythemia, thrombotic thrombocytopenic purpura, severe anemia and embolism at distant sites, leading to infarction of the optic nerve. None of these diseases, except diabetes mellitus, was present in our patient.

Studies have shown that blindness can follow drug-induced hypotension, but these cases are more frequent in association with the factors cited above or with anemia, excessive venous infusion or variations in the neural microcirculation. We believe that the periods of hypotension affecting our patient, together with diabetes and thrombosis of a jugular vein, were the factors that triggered his blindness.

Blindness has also been reported to be a consequence of increased intraocular pressure due to the compression exerted by eye protectors during the intraoperative period.^6^ These protectors do not permit eyelid expansion when edema occurs and the increased pressure is transmitted to the ocular globe. This type of protector was not used for our patient.

Depending on the site of the lesion, anterior ischemic optic neuropathy may be observed as well as posterior ischemic optic neuropathy. The presence of a disorder of visual acuity and/or of a defect of the visual field is necessary for the diagnosis of anterior ischemic optic neuropathy, in addition to edema of the optic disk followed by complete or sectorial optic atrophy.^7^ The changes observed by fundoscopy (cloudy papillae and edema of the optic disk) and the alteration of visual acuity detected in our patient agree with a diagnosis of anterior ischemic optic neuropathy. This probably occurred because of intraoperative hypotension of about 40 minutes duration, with a possible contribution by decompensated diabetes mellitus and thrombosis of the right-side internal jugular vein.

Thus, during the preoperative period it is the surgeon's obligation to control or reduce risk factors such as anemia, systemic arterial hypertension and diabetes, and to avoid intraoperative hypotension. In addition, the patient's head must be kept elevated during the postoperative period in order to prevent the occurrence of this rare but debilitating complication. In situations where the patient complains of visual deficit during the postoperative period, an immediate evaluation by an ophthalmologist is of fundamental importance for the indication of specific procedures such as the use of intraocular hypotensive agents and systemic mannitol and corticoids.^7^
